# Curious Application of the Chloruret of Lime

**Published:** 1826-07

**Authors:** 


30. *Chloruret of Lime.* We have more than once drawn the attention of our countrymen to this disinfecting agent, which is of very easy formation, being nothing more than a suspension of lime, in water saturated with chlorine. An ounce of this saturated solution is sufficient for a gallon of water, and is employed on the Continent, with great advantage, where bodies are to be handled that are highly putrified, both in judicial and common anatomical proceedings. A curious application of this substance was lately made in France, in the case of a retained placenta, which became very putrid, and disengaged the most disgusting effluvia producing very unpleasant symptoms. M. Delandes threw up injections composed of chloruret of lime in decoction of marsh-mallows, a drachm to a pint. The fetid odour ceased almost entirely on the first application, and was completely subdued by the second. After this the acute pains, with which the patient had been affected, were relieved. The remains of the placenta were not quite expelled till 18 days after the birth of the child; but during its separation the putrefactive smell did not return.—*Rev. Med.*

